# Principal components-based selection criteria for genetic improvement of growth in sheep breeding programs

**DOI:** 10.1186/s12711-025-00992-0

**Published:** 2025-09-25

**Authors:** Ajoy Mandal, Indrajit Gayari, Sylvia Lalhmingmawii, David R. Notter, Hasan Baneh

**Affiliations:** 1 Animal Breeding Section, ICAR-National Dairy Research Institute, Eastern Regional Station, Nadia, Kalyani, West Bengal 741235 India; 2 School of Animal Sciences, Virginia Tech, Blacksburg, VA 24061 USA; 3 Project Center for Agro Technologies, Skolkovo Institute of Science and Technology (Skoltech), Moscow, 121205 Russia; 4 Animal Science Research Department, Kurdistan Agricultural and Natural Resources Research and Education Center, Agricultural Research, Education and Extension Organization (AREEO), Sanandaj, Iran

## Abstract

**Background:**

The objective of this study was to investigate the use of principal components (PC) as potential selection criteria to improve growth in sheep. The PC were derived from body weights of 2223 Muzaffarnagari lambs at birth, 90, 180, 270 and 360 days of age. Univariate animal models including various combinations of direct and maternal effects were fitted to the PC. Genetic correlations among PC and with body weights and estimated growth curve parameters for the Brody and Richards functions were estimated using bivariate animal models.

**Results:**

The first three PC explained 94% of multivariate variation in body weights. PC1 contrasted lambs with larger versus smaller body weights at all postnatal ages. PC2 contrasted lambs with heavier versus lighter birth weights, with little emphasis on postnatal weights. PC3 placed positive emphasis on weights at birth and after 6 months of age but negative emphasis on weight at 3 through 9 months of age. Direct heritabilities for PC1, PC2, and PC3 were 0.19, 0.12 and 0.08, respectively. Maternal genetic and permanent environmental effects affected PC1 (0.04 and 0.08, respectively). PC2 was influenced by maternal genetic effects (0.10). Direct genetic correlations of PC1 with PC2 and PC3 were 0.48 and 0.72. The maternal genetic correlation between PC1 and PC2 was 0.97. Genetic relationships of PC1 with yearling weight and with estimates of final body weight from both growth functions exceeded 0.65. PC2 was genetically correlated with birth weight (≥ 0.64) and degree of maturity for body weight at birth (u_0_; ≥ 0.83). PC3 had negative genetic correlations with measures of maturing rate (~ -0.86) and with u_0_ ( -0.52 and -0.49), but positive correlations with final body weight (0.85 and 0.90) and time required to reach 50% of mature weight (0.83). Maternal genetic correlations of PC1 and PC2 with birth weight and u_0_ exceeded 0.83.

**Conclusions:**

We conclude that PC could be used as selection criteria in genetic improvement programs in sheep. Also, selection on PC1 and PC2 would likely be adequate to describe and improve direct and maternal genetic potentials for postnatal growth and birth weight, respectively, in Muzaffarnagari lambs.

**Supplementary Information:**

The online version contains supplementary material available at 10.1186/s12711-025-00992-0.

## Background

Growth, which is defined as an increase in live weight as animals age, is one of the most important characteristics for meat production systems [1, 2]. The economically valuable traits like mature weight, mature age, and rate of maturing at different phases of growth can affect the growth rate [2, 3]. Therefore, the variation of growth in a population can be observed in both variation in the overall growth rate and in the shape of the growth curve. Growth in mammals is a complex phenomenon which is affected by genetic and environmental effects arising from both individual and maternal influences [4–6]. However, maternal effects are more important at early ages and decline in importance with increasing lamb age, as the lambs become independent of the mother [6–8].

Growth-related phenotypes in sheep are recorded as body weights at different ages, usually at birth, weaning, and then at regular intervals until maturity. These traits can be directly used in a breeding program to evaluate the genetic potential of the lambs for each trait, or to describe the body weight changes over age using three-parameter (e.g., logistic, Brody, Gompertz and Von Bertalanffy) or four-parameter (e.g., Richards) growth curves. These mathematical growth functions explain weight-age relationship using a few biologically meaningful parameters [2, 9]. The estimated growth curve parameters can be used to evaluate nutritional practices and optimal slaughter ages, describe interactions among subpopulations or treatments and time, identify heavier animals at younger ages, and evaluate correlated responses to selection [1, 10–12]. Estimates of growth-curve parameters may also be used as selection criteria to alter the relationship between body weight and age [13, 14].

Some studies have shown that growth curve parameters in sheep are heritable [1, 3, 14–17] and, therefore, could be used in a selective breeding program to modify the growth curve. However, our previous work on Muzaffarnagari sheep [2] showed that parameters associated with growth rates from birth to yearling for both Brody and Richards functions were relatively lowly heritable. In addition, none of the growth curve parameters were significantly influenced by maternal effects in this breed. Limited direct and maternal genetic variation and sensitivity to environmental factors may therefore limit the practical use of growth-curve parameter estimates as reliable selection criteria in breeding programs. There appears to be no obvious superiority to using these parameter estimates in selection when compared with multi-trait genetic evaluations of individual body weights [2].

Multi-trait analysis uses correlations among the traits to account for missing records and provides a relatively good depiction of the realized growth trajectory. However, computational demands increase rapidly when the number of weights increases. Multivariate statistical techniques like principal component analysis (PCA) have gained prominence in livestock research. PCA transforms correlated traits, such as body weights measured at different ages, into a set of uncorrelated principal components that capture the major sources of variation of growth. This approach provides a powerful framework for dissecting growth traits, as it enables the identification of growth patterns and relationships that may not be reflected by traditional methods. The principal components are empirical and data-driven, making them less reliant on predefined mathematical assumptions about growth trajectories, but are correspondingly less amenable to interpretation in terms of underlying growth curves. These features motivated us to investigate if applying PCA to longitudinal body weights would fill critical knowledge gaps and consider if principal components could be used as selection criteria in sheep breeding programs.

PCA has been used in animal breeding and genetics to reduce the size of the direct additive genetic covariance matrix in multiple-trait models, study relationships among predicted breeding values, develop descriptive multiple-trait indexes [18–20], and explain population and family structures from genome-wide association studies where the number of potential marker effects often exceeds the number of experimental units [21]. We did not, however, identify previous studies that critically compared PCA and classical growth-curve analysis in sheep. Therefore, the objectives of the current study were to (1) investigate the use of PCA-derived components to provide a data-driven perspective on growth dynamics, (2) estimate the genetic and phenotypic parameters of these components to identify potential selection criteria for breeding programs, and (3) evaluate genetic relationship of the components with traditional growth-curve parameters to evaluate their utility, limitations, and robustness in the genetic improvement of growth in Muzaffarnagari sheep.

## Methods

### Flock management

Data used for this study were body weight records and pedigree information from an experimental breeding flock of Muzaffarnagari sheep collected over 29 years at the Central Institute for Research on Goats, Makhdoom, Mathura, Uttar Pradesh, India. The flock was established in 1976 under the “All India Coordinated Research Project (AICRP) on Sheep Breeding for Mutton Production”. The farm is located at 27º 10′ N, 78º 02′ E, and 169 m above sea level. The climate of this area is semi-arid. Temperatures range from 0 °C to 45 °C, and the annual precipitation of about 750 mm occurs mainly during the monsoon from July to September. The flock is maintained under semi-intensive feeding management. Ewes receive 250 g of a concentrate diet daily, are allowed 6 to 7 h of grazing, and have ad libitum access to dry and green fodder. Controlled mating was practiced in the flock. Ewes were first exposed to rams at 12 to 14 months of age. Each breeding ram was allowed to mate with 20 to 25 ewes, and ewes were mated twice at each estrus. Rams were used for breeding for about 3 years. Breeding occurred in May and June and in October and November, with lambing in October and November and in March and April, respectively. Lambs were weighed and ear tagged at birth, and the date of birth, sex, birth type and sire and dam were recorded. Regular health monitoring and treatment were applied, including vaccinations against enterotoxaemia, foot and mouth disease, sheep pox, haemorrhagic septicemia, and peste des petits ruminants. Characteristics of the breed and typical husbandry practices were described by Mandal et al. [22], and additional details about data collection procedures were provided by Mandal et al. [23].

### Data and principal component analysis

Body weights of 2611 Muzaffarnagari lambs recorded at birth, 90, 180, 270 and 360 days of age were used. Lambs with atypical growth patterns were removed (n = 7) [23]. Because complete data (without missing values) for all the variables is required for principal component analysis, only 2223 lambs with all five body weight records were retained for subsequent analyses.

Body weights at different ages had different scales and were affected by different environmental factors. Weights were therefore expressed as deviations from the overall mean of each weight and adjusted for environmental effects including birth year, birth season (spring and autumn), lamb sex, birth type (single or twin), and dam parity (five classes) such that

1 $$\text{y}^* =\text{ y}- (\upmu +\text{ BY }+\text{ BS }+\text{ S }+\text{ LS }+\text{ DP}),$$

where y* is the adjusted weight; y is the actual weight; μ is the overall mean weight; and BY, BS, S, LS, and DP are estimated effects of birth year, birth season, sex, litter size and parity of dam, respectively. Estimates of fixed effects were derived for each weight using univariate fixed-effect models in PROC GLM of SAS (SAS Inst., Cary, NC). Descriptive statistics for adjusted body weights at different ages are shown in Table 1. Principal component analysis was performed on adjusted weights using the PRINCOMP procedure in SAS. Outliers with principal component (PC) values that differed from the mean by greater than 3.35 standard deviations (SD) for one or more of the PC (13 lambs; one, four and eight lambs for PC1, PC2, and PC3, respectively) were identified using an iterative approach [2] and removed from the data, leaving 2210 animals for genetic analysis. Descriptive statistics for PC1 through 5 are shown in Table 2.

**Table 1:** Descriptive statistics for the adjusted weights

Trait	Number	Minimum	Maximum	Mean	SD
BW	2223	-2.14	1.87	0.00	0.55
W90	2223	-9.56	9.71	0.00	2.99
W180	2223	-13.51	18.86	0.00	3.82
W270	2223	-13.69	14.31	0.00	3.94
W360	2223	-18.96	14.94	0.00	4.22

BW, W90, W180, W270 and W360 are body weights (kg) at birth, 90, 180, 270 and 360 days of age adjusted for overall mean and environmental effects, respectively. SD: standard deviation

**Table 2:** Descriptive statistics for the principal components

Trait	Number	Minimum	Maximum	Mean	SD
PC1	2210	-6.11	6.07	0.00	1.87
PC2	2210	-2.94	2.86	0.00	0.88
PC3	2210	-2.02	2.00	0.00	0.62
PC4	2210	-2.37	1.96	0.00	0.44
PC5	2210	-1.94	1.27	0.00	0.32

PC: principal components, SD: standard deviation

The proportion of the variance in the body weights from birth to 360 days of age that was explained by each PC (PEV_i_) was estimated as:

2 $${PEV}_{i}=\frac{{egv}_{i}}{\sum_{i=1}^{5}{egv}_{i}},$$

where egv_i_ is the eigenvalue associated with the i^th^ PC.

To investigate the consistency of the estimated PC, the PC analysis was subsequently performed separately for animals of different sexes and those born in different birth seasons. To do this, the aforementioned linear model was fitted on the data while the factor of interest (birth season or sex) was excluded from the model.

### Genetic analysis

(Co)variance components and genetic parameters for the first three PC were estimated using six different univariate mixed linear animal models:

3 $$\text{Model }1\;\mathbf{y} = \mathbf{1}\upmu + {\mathbf{Z}}_{1}\mathbf{a} + \mathbf{e}$$

4 $$\text{Model }2\; \mathbf{y} = \mathbf{1}\upmu + {\mathbf{Z}}_{1}\mathbf{a} + {\mathbf{Z}}_{2}\mathbf{c} + \mathbf{e}$$

5 $$\text{Model }3 \;\mathbf{y} = \mathbf{1}\upmu + {\mathbf{Z}}_{1}\mathbf{a} + {\mathbf{Z}}_{3}\mathbf{m} + \mathbf{e} \quad \text{Cov }(\text{a},\text{ m}) = 0$$

6 $$\text{Model }4 \;\mathbf{y} = \mathbf{1}\upmu + {\mathbf{Z}}_{1}\mathbf{a} + {\mathbf{Z}}_{3}\mathbf{m} + \mathbf{e} \quad \text{Cov }(\text{a},\text{ m}) =\mathbf{A}{\sigma }_{a,m}$$

7 $$\text{Model }5 \;\mathbf{y} = \mathbf{1}\upmu + {\mathbf{Z}}_{1}\mathbf{a} + {\mathbf{Z}}_{2}\mathbf{c} + {\mathbf{Z}}_{3}\mathbf{m} + \mathbf{e} \quad \text{Cov }(\text{a},\text{ m}) = 0$$

8 $$\text{Model }6 \;\mathbf{y} = \mathbf{1}\upmu + {\mathbf{Z}}_{1}\mathbf{a} + {\mathbf{Z}}_{2}\mathbf{c} + {\mathbf{Z}}_{3}\mathbf{m} + \mathbf{e} \quad \text{Cov }(\text{a},\text{ m}) =\mathbf{A}{\sigma }_{a,m}$$

where **y** is vector of values for each PC (with dimensions of 2210×1); μ is the overall mean; **a, c**, **m** and **e** are vectors of direct additive genetic (with dimensions 2591×1), maternal permanent environmental (with dimensions 1215×1), maternal additive genetic (with dimensions of 1423×1), and residual effects (with dimensions 2210×1), respectively; **1** is a vector of ones (with dimensions of 2210×1); **Z**_**1**_ (with dimensions 2210×2591), **Z**_**2**_ (with dimensions 2210×1215), and **Z**_**3**_ (with dimensions 2210×1423) are incidence matrices relating phenotypes to fixed, direct additive genetic, dam permanent environmental, and maternal additive genetic effects, respectively. Direct additive genetic, maternal permanent environmental, maternal additive genetic, and residual effects were assumed to be normally distributed with means of zero and variances of ${\mathbf{A}\sigma }_{a}^{2}$, ${\mathbf{I}}_{\mathbf{c}}{\sigma }_{c}^{2}$, ${\mathbf{A}\sigma }_{m}^{2}$, and ${\mathbf{I}}_{\mathbf{n}}{\sigma }_{e}^{2}$, respectively, where ${\sigma }_{a}^{2}$, ${\sigma }_{m}^{2}$, ${\sigma }_{c}^{2}$ and ${\sigma }_{e}^{2}$ are direct additive genetic, maternal additive genetic, maternal permanent environmental, and residual variances, respectively. **A** is the numerator relationship matrix (with dimensions 2859 × 2859) containing additive genetic covariances among animals computed using pedigree information, ${\mathbf{I}}_{\mathbf{c}}$ and ${\mathbf{I}}_{\mathbf{n}}$ are identity matrices with dimensions equal to numbers of dams (c = 1215) and records (n = 2210), respectively. In Model 4 and 6, a covariance between additive direct and maternal genetic effects of $\mathbf{A}{\sigma }_{a,m}$, was assumed where ${\sigma }_{a,m}$ is the direct-maternal genetic covariance. Likelihood ratio tests (LRT) were applied to determine the best model for each PC (P < 0.05).

Genetic parameters including heritability (${h}^{2}$), maternal heritability (${m}^{2}$), maternal permanent environmental effects (${c}^{2}$), and direct-maternal genetic correlation (${r}_{a,m}$) were calculated as ratios of estimated (co)variance components, where:

$${h}^{2}=\frac{{\sigma }_{a}^{2}}{{\sigma }_{p}^{2}}; {m}^{2}=\frac{{\sigma }_{m}^{2}}{{\sigma }_{p}^{2}};{c}^{2}=\frac{{\sigma }_{c}^{2}}{{\sigma }_{p}^{2}};{r}_{a,m}=\frac{{\sigma }_{a,m}}{\sqrt{{\sigma }_{a}^{2}*{\sigma }_{m}^{2}}};$$

and ${\sigma }_{p}^{2}$ is the phenotypic variance. Bivariate mixed linear animal models were used to estimate (co)variances and correlations of random effects among PC and between PC and body weights at birth (BW) and yearling age (W360). To address the possibility of utilizing PC of body weights as an alternative to estimates of growth curve parameters, estimates of growth curve parameters from Mandal et al. [23] were used. In that study, body weights at birth, 3, 6, 9, and 12 months were used to fit several growth functions. The Brody and Richards functions were selected as the best 3- and 4- parameters growth curves, respectively, for this breed. Therefore, only estimated growth curve parameters for these two functions were used in this study, which included a predicted asymptotic final weight (A; kg) and a maturing-rate parameter (k). Additional derived parameters included u_0_ (the degree of maturity (BW/A) at birth), t_50_ (the time in days required to reach 50% of A), and, for the Richards function, u_I_ (the predicted degree of maturity associated with the maximum postnatal growth rate). Definition, estimation, and interpretation of these parameters were previously described and discussed [2, 23]. Bivariate animal models used to estimate covariances between PCs and estimated growth-curve parameters were:

$$\left(\begin{array}{c}{\mathbf{y}}_{\mathbf{i}}\\ {\mathbf{y}}_{\mathbf{j}}\end{array}\right)=\left(\begin{array}{cc}{\mathbf{X}}_{\mathbf{i}}& 0\\ 0& {\mathbf{X}}_{\mathbf{j}}\end{array}\right)\left(\begin{array}{c}{\mathbf{b}}_{\mathbf{i}}\\ {\mathbf{b}}_{\mathbf{j}}\end{array}\right)+\left(\begin{array}{cc}{\mathbf{Z}}_{{1}_{\mathbf{i}}}& 0\\ 0& {\mathbf{Z}}_{{1}_{\mathbf{j}}}\end{array}\right)\left(\begin{array}{c}{\mathbf{a}}_{\mathbf{i}}\\ {\mathbf{a}}_{\mathbf{j}}\end{array}\right)+\left(\begin{array}{cc}{\mathbf{Z}}_{{2}_{\mathbf{i}}}& 0\\ 0& {\mathbf{Z}}_{{2}_{\mathbf{j}}}\end{array}\right)\left(\begin{array}{c}{\mathbf{c}}_{\mathbf{i}}\\ {\mathbf{c}}_{\mathbf{j}}\end{array}\right)+\left(\begin{array}{cc}{\mathbf{Z}}_{{3}_{\mathbf{i}}}& 0\\ 0& {\mathbf{Z}}_{{3}_{\mathbf{j}}}\end{array}\right)\left(\begin{array}{c}{\mathbf{m}}_{\mathbf{i}}\\ {\mathbf{m}}_{\mathbf{j}}\end{array}\right)+\left(\begin{array}{c}{\mathbf{e}}_{\mathbf{i}}\\ {\mathbf{e}}_{\mathbf{j}}\end{array}\right),$$

With ${\sigma }_{a,m}$ = 0. Subscript i and j pertains to the traits 1 and 2 respectively. The models further assumed:

$$\left(\begin{array}{c}\left(\begin{array}{c}{\mathbf{a}}_{\mathbf{i}}\\ {\mathbf{a}}_{\mathbf{j}}\end{array}\right)\\ \left(\begin{array}{c}{\mathbf{c}}_{\mathbf{i}}\\ {\mathbf{c}}_{\mathbf{j}}\end{array}\right)\\ \left(\begin{array}{c}{\mathbf{m}}_{\mathbf{i}}\\ {\mathbf{m}}_{\mathbf{j}}\end{array}\right)\\ \left(\begin{array}{c}{\mathbf{e}}_{\mathbf{i}}\\ {\mathbf{e}}_{\mathbf{j}}\end{array}\right)\end{array}\right)\sim N\left(0, \left[\begin{array}{cccccccc}{\mathbf{A}\upsigma }_{{\text{a}}_{\text{i}}}^{2}& \mathbf{A}{\upsigma }_{{\text{a}}_{\text{i},\text{j}}}& 0& 0& 0& 0& 0& 0\\ \mathbf{A}{\upsigma }_{{\text{a}}_{\text{j},\text{i}}}& {\mathbf{A}\upsigma }_{{\text{a}}_{\text{j}}}^{2}& 0& 0& 0& 0& 0& 0\\ 0& 0& {{\mathbf{I}}_{\mathbf{c}}\upsigma }_{{\text{c}}_{\text{i}}}^{2}& {\mathbf{I}}_{\mathbf{c}}{\upsigma }_{{\text{c}}_{\text{i},\text{j}}}& 0& 0& 0& 0\\ 0& 0& {\mathbf{I}}_{\mathbf{c}}{\upsigma }_{{\text{c}}_{\text{j},\text{i}}}& {{\mathbf{I}}_{\mathbf{c}}\upsigma }_{{\text{c}}_{\text{j}}}^{2}& 0& 0& 0& 0\\ 0& 0& 0& 0& {\mathbf{A}\upsigma }_{{\text{m}}_{\text{i}}}^{2}& \mathbf{A}{\upsigma }_{{\text{m}}_{\text{i},\text{j}}}& 0& 0\\ 0& 0& 0& 0& \mathbf{A}{\upsigma }_{{\text{m}}_{\text{j},\text{i}}}& {\mathbf{A}\upsigma }_{{\text{m}}_{\text{j}}}^{2}& 0& 0\\ 0& 0& 0& 0& 0& 0& {{\mathbf{I}}_{\mathbf{n}}\upsigma }_{{\text{e}}_{\text{i}}}^{2}& {\mathbf{I}}_{\mathbf{n}}{\upsigma }_{{\text{e}}_{\text{i},\text{j}}}\\ 0& 0& 0& 0& 0& 0& {\mathbf{I}}_{\mathbf{n}}{\upsigma }_{{\text{e}}_{\text{j},\text{i}}}& {{\mathbf{I}}_{\mathbf{n}}\upsigma }_{{\text{e}}_{\text{j}}}^{2}\end{array}\right]\right)$$

where ${\sigma }_{{a}_{i,j}}={\sigma }_{{a}_{j,i}}$, ${\sigma }_{{c}_{i,j}}={\sigma }_{{c}_{j,i}}$, ${\sigma }_{{m}_{i,j}}={\sigma }_{{m}_{j,i}}$ and ${\sigma }_{{e}_{i,j}}={\sigma }_{{e}_{j,i}}$ are direct additive genetic, dam permanent environmental, maternal additive genetic and residual covariances, respectively, between traits i and j. The genetic analysis was performed using an AIREML algorithm implemented in WOMBAT [24] under the Linux operation system. The convergence criterion was 10^−6^.

## Results

Descriptive statistics for adjusted weights (Table 1) confirmed that the values were distributed around a mean of zero, with an increasing trend in SD from birth (0.55 kg) to 360 days of age (4.22 kg). Phenotypic correlations (r_p_) among weights were positive, ranging from 0.34 between birth and yearling weights to 0.87 between weights at 270 and 360 days (Table 3). Correlations among weights increased with age and as the time between weighing decreased. Birth weight had a relatively weak correlation with all subsequent weights, ranging from 0.42 at 90 days to 0.34 for yearling weight.

**Table 3:** Phenotypic Pearson correlations between adjusted traits for the entire dataset and for different sexes and birth seasons

Trait 1	Trait 2	Entire dataset	Lamb sex		Birth season	
			Male	Female	March–April	October–November
BW	W90	0.42	0.37	0.43	0.46	0.38
BW	W180	0.36	0.30	0.37	0.41	0.32
BW	W270	0.36	0.33	0.37	0.39	0.34
BW	W360	0.34	0.32	0.35	0.39	0.34
W90	W180	0.78	0.80	0.78	0.78	0.79
W90	W270	0.71	0.74	0.71	0.68	0.73
W90	W360	0.63	0.65	0.63	0.64	0.66
W180	W270	0.83	0.87	0.82	0.84	0.86
W180	W360	0.73	0.78	0.71	0.77	0.80
W270	W360	0.87	0.88	0.87	0.89	0.88

BW, W90, W180, W270 and W360 are body weights (kg) at birth, 90, 180, 270 and 360 days of age, respectively, and were expressed as deviations from the overall means for each body weight

**Fig. 1:**
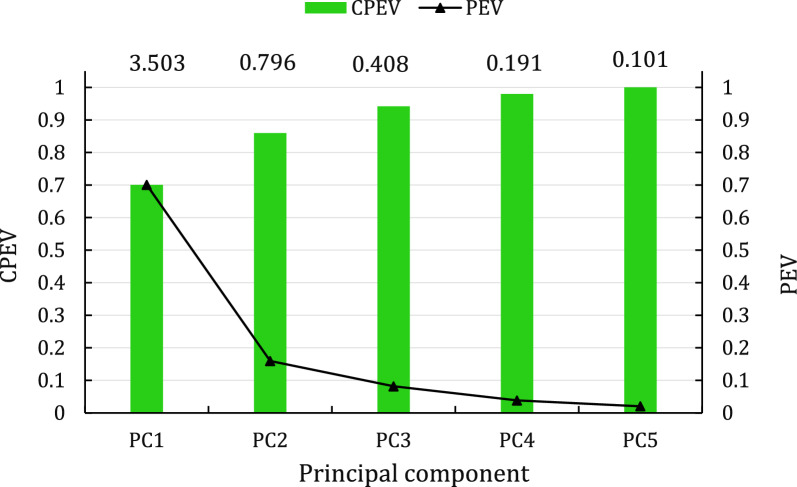
Cumulative proportion of explained variance (CPEV) and proportion of explained variance (PEV) of the principal components for body weights from birth to 12 months of age in Muzaffarnagari sheep. Eigenvalues, which reflect the importance of each principal component in explaining the multivariate variation in body weight, are shown at the top of each column

Descriptive statistics for PC (Table 2) indicated that the PC were normally distributed around a mean of zero. As expected, PC1 had the greatest range (-6.11 to 6.07) and SD (1.87), and PC5 had the lowest range (-1.94 to 1.27) and SD (0.32). Individual and cumulative proportions of the multivariate variance explained by each PC are shown in Fig. 1. The first, second and third PC explained 70, 16 and 8% of the variation, respectively. Results of the PC analyses for different lamb sexes and birth seasons indicated high levels of consistency between PC for different classes (See Additional file 1: Figure S1 and ﻿Additional file 2: Figure S2). For all four subsets (males, females, and birth season 1 and 2), the first three eigenvalues had values of 3.5, 0.8 and 0.4, respectively, which were the same as those obtained for the whole dataset. In total, 94% of the total multivariate variation in body weight from birth to 12 months of age in this breed was explained by the first three components. We therefore used only the first three PC in subsequent analyses.

**Fig. 2:**
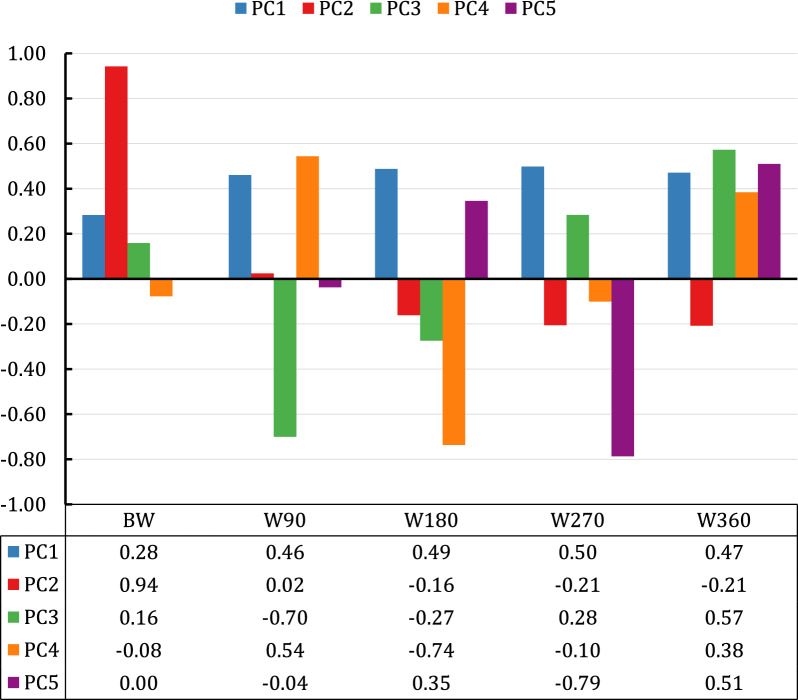
Eigenvectors of principal components (PC) 1 to 5 for residual body weighs at birth (BW) and at 90 (W90), 180 (W180), 270 (W270), and 360 (W360) days of age

Eigenvectors for each PC are shown in Fig. 2. Eigenvector weightings for PC1 were positive for all body weights. Weightings ranged from 0.46 to 0.50 for postnatal weights, and were almost double the weight of 0.28 assigned to birth weight. The second PC emphasized birth weight. The eigenvector weighting for birth weight in PC2 was 0.94, with modest negative emphasis on weights recorded at ≥ 6 months of age. PC3 placed strong negative emphasis on 90-day weaning weight and modest negative emphasis on 180-day weight, but modest positive emphasis on weights at birth and at 270 days of age and relatively high positive (eigenvector = 0.57) emphasis on yearling weight. As expected, Pearson correlations between the PC and the adjusted phenotypes were consistent with the eigenvectors (See Additional file 3: Figure S3). Results of the PC analyses for different sexes and birth seasons (See Additional file 4: Figure S4 and Additional file 5: Figure S5, respectively) were similar to those obtained for the whole population.

Estimates of (co)variance components and associated genetic parameters for the first three PC are summarized in Table 4. For PC1, including maternal effects improved goodness of fit, but reduced the estimate of direct heritability (h^2^) from 0.27 for Model 1 to 0.22 and 0.18 for models with maternal permanent environmental effects (c^2^; Model 2) or additive maternal effects (m^2^; Model 3), respectively. Model 4 yielded a relatively large positive estimate of the additive direct-maternal covariance and was superior to Model 3 but did not differ in LogL from Model 5. Model 6 was somewhat superior to Model 5 in goodness of fit, but the estimate of r_a,m_ from Model 6 was unreasonably large (0.99). Estimates of h^2^ for models that included an estimate of σ_a,m_ were correspondingly reduced to 0.13 for Model 4 and 0.14 for Model 6. Based on LRT, fitting both additive genetic and permanent environmental maternal effects (Model 5) significantly increased goodness of fit compared to either Model 3 or Model 4 and was selected as the best model for PC1. The estimate of h^2^ for PC1 in Model 5 was moderate (0.19), and the total maternal effect (m^2^ + c^2^) accounted for 12% of the phenotypic variance. The maternal permanent environmental effect (c^2^ = 0.08) was larger than the additive maternal effect (m^2^ = 0.04).

**Table 4:** Co-variance components and corresponding genetic parameter estimates for PC1, PC2 and PC3

**Trait**	**Model**	${{\varvec{\sigma}}}_{{\varvec{a}}}^{2}$	${{\varvec{\sigma}}}_{{\varvec{m}}}^{2}$	${{\varvec{\sigma}}}_{{\varvec{pe}}}^{2}$	${{\varvec{\sigma}}}_{{\varvec{a}},{\varvec{m}}}$	${{\varvec{\sigma}}}_{{\varvec{e}}}^{2}$	${{\varvec{\sigma}}}_{{\varvec{p}}}^{2}$	${{\varvec{h}}}^{2}$	${{\varvec{c}}}^{2}$	${{\varvec{m}}}^{2}$	${{\varvec{r}}}_{{\varvec{a}},{\varvec{m}}}$	${1-{\varvec{e}}}^{2}$	**LogL**
PC1	1	0.9668				2.6401	3.6069	0.27 ± 0.05				0.27	-2459.397
	2	0.7825		0.3910		2.4042	3.5776	0.22 ± 0.05	0.11 ± 0.03			0.33	-2452.007
	3	0.6393	0.3558			2.6005	3.5957	0.18 ± 0.05		0.10 ± 0.03		0.28	-2453.192
	4	0.4705	0.2112		0.2183	2.7043	3.6043	0.13 ± 0.04		0.06 ± 0.03	0.69 ± 0.42	0.25	-2451.319
	**5**	**0.6949**	**0.1518**	**0.2770**		**2.4536**	**3.5772**	**0.19 ± 0.05**	**0.08 ± 0.04**	**0.04 ± 0.03**		**0.31**	**-2451.140**
	6	0.5045	0.0730	0.2525	0.1906	2.5633	3.5837	0.14 ± 0.05	0.07 ± 0.04	0.02 ± 0.03	0.99 ± 0.93	0.28	-2449.340
PC2	1	0.1582				0.6338	0.7920	0.20 ± 0.04				0.20	-807.022
	2	0.1262		0.0655		0.5948	0.7866	0.16 ± 0.04	0.08 ± 0.03			0.24	-802.163
	**3**	**0.0968**	**0.0763**			**0.6162**	**0.7893**	**0.12 ± 0.04**		**0.10 ± 0.03**		**0.22**	**-796.677**
	4	0.1132	0.0925		-0.0236	0.6060	0.7882	0.14 ± 0.05		0.12 ± 0.04	-0.23 ± 0.21	0.23	-796.141
	5	0.0980	0.0656	0.0174		0.6070	0.7881	0.12 ± 0.04	0.02 ± 0.03	0.08 ± 0.03		0.23	-796.444
	6	0.1158	0.0800	0.0185	-0.0233	0.5960	0.7871	0.15 ± 0.05	0.02 ± 0.03	0.10 ± 0.04	-0.24 ± 0.21	0.24	-795.891
PC3	**1**	**0.0315**				**0.3594**	**0.3909**	**0.08 ± 0.03**				**0.08**	**-57.396**
	2	0.0292		0.0114		0.3502	0.3907	0.08 ± 0.03	0.03 ± 0.03			0.10	-56.807
	3	0.0289	0.0041			0.3579	0.3908	0.07 ± 0.03		0.01 ± 0.02		0.08	-57.158
	4	0.0500	0.0171		-0.0188	0.3437	0.3921	0.13 ± 0.05		0.04 ± 0.03	-0.64 ± 0.21	0.12	-56.157
	5	0.0287	1.105E-05	0.0103		0.3506	0.3907	0.07 ± 0.03	0.03 ± 0.03	0.003 ± 0.02		0.10	-56.792
	6	0.0499	0.0132	0.0101	-0.7921	0.3368	0.3920	0.13 ± 0.05	0.03 ± 0.03	0.03 ± 0.03	-0.70 ± 0.24	0.14	-55.821

${\sigma }_{a}^{2}$: direct additive genetic variance; ${\sigma }_{m}^{2}$: maternal additive genetic variance; ${\sigma }_{pe}^{2}$: maternal permanent environmental variance; ${\sigma }_{a,m}$: direct-maternal additive genetic covariance; ${\sigma }_{e}^{2}$: residual variance; ${\sigma }_{p}^{2}$: phenotypic variance; ${h}^{2}$: direct heritability; ${c}^{2}$: proportion of maternal permanent environmental variance to phenotypic variance; ${m}^{2}$: maternal heritability; ${r}_{a,m}$: direct-maternal genetic correlation; ${1-e}^{2}$: the total proportion of total variance explained by the model; LogL: logarithm of the likelihood function. The best model for each PC is shown in bold

For PC2, fitting maternal permanent environmental effects in Model 2 (c^2^ = 0.08) or additive maternal effects in Model 3 (m^2^ = 0.10) both significantly improved goodness of fit compared to Model 1. Attempting to partition maternal effect into additive and permanent environmental effects in Model 5 (c^2^ = 0.02; m^2^ = 0.08) resulted in no improvement in likelihood compared to Model 3. Estimates of r_a,m_ from Models 4 and 6 were relatively small and negative, and fitting σ_a,m_ did not improve goodness of fit relative to that achieved by Models 3 and 5, respectively. Model 3 was therefore considered to be the best model for PC2, with estimates of h^2^ and m^2^ of 0.12 and 0.10, respectively.

Based on LRT, only direct genetic effects significantly affected PC3. Models 2, 3, and 5 indicated that maternal effects accounted for, at most, 3% of phenotypic variance. Model 1 was therefore chosen as the best model for PC3, with an estimate of h^2^ of 0.08.

Direct genetic, maternal genetic, residual and phenotypic correlations estimates using bivariate analysis among PC are shown in Table 5. As expected, PC were not correlated phenotypically (${r}_{p}\cong 0$), but their genetic correlations were consistently positive, ranging from 0.19 between PC2 and PC3 to 0.72 between PC1 and PC3. Direct genetic effects on PC1 and PC2 were moderately correlated (0.48), and their maternal genetic correlation was close to unity (0.97).

**Table 5:** Estimates of heritability, direct genetic, maternal genetic, residual and phenotypic correlation among principal components, fitting best model for each trait using bivariate analysis

**Trait 1**	**Trait 2**	**Fitted models**	${{\varvec{h}}}_{1}^{2}$	${{\varvec{h}}}_{2}^{2}$	${{\varvec{c}}}_{1}^{2}$	${{\varvec{m}}}_{1}^{2}$	${{\varvec{m}}}_{2}^{2}$	${{\varvec{r}}}_{{\varvec{a}}}$	${{\varvec{r}}}_{{\varvec{m}}}$	${{\varvec{r}}}_{{\varvec{e}}}$	${{\varvec{r}}}_{{\varvec{p}}}$
PC1	PC2	5, 3	0.16 ± 0.04	0.12 ± 0.04	0.06 ± 0.04	0.07 ± 0.03	0.10 ± 0.03	0.48 ± 0.19	0.97 ± 0.22	-0.17 ± 0.04	0.02 ± 0.02
PC1	PC3	5, 1	0.19 ± 0.05	0.09 ± 0.03	0.08 ± 0.04	0.05 ± 0.03		0.72 ± 0.18		-0.11 ± 0.03	0.01 ± 0.02
PC2	PC3	3, 1	0.13 ± 0.04	0.08 ± 0.03		0.10 ± 0.03		0.19 ± 0.24		-0.03 ± 0.03	0.00 ± 0.02

Fitted models are the models from Table 4 fitted for each of the trait 1 and 2, respectively; ${h}_{1}^{2}$ and ${h}_{2}^{2}$: direct heritabilities of the trait 1 and 2, respectively; ${c}_{1}^{2}$: proportion of maternal permanent environmental variance to phenotypic variance for PC1; ${m}_{1}^{2}$ and ${m}_{2}^{2}$: maternal heritabilities for the trait 1 and 2, respectively; ${r}_{a}$: direct genetic correlation; ${r}_{m}$: maternal genetic correlation; ${r}_{e}$: residual correlation; ${r}_{p}$: phenotypic correlation

All of the PC were genetically positively correlated with birth and yearling weights (Table 6). PC1 had a genetic correlation with yearling weight of 0.65 and a genetic correlation with birth weight of 0.37. In contrast, PC2 had a strong positive genetic correlation with birth weight (0.64) but a weaker correlation with yearling weight (0.30). Genetic correlations of PC3 with birth and yearling weights were also positive but modest (0.31 and 0.37, respectively). Birth weight also had a strong maternal genetic correlation with PC1 (0.83) and PC2 (0.94). The residual correlations among the PCs and body weights were also generally positive, except for the correlation of -0.26 between PC2 and yearling weight.

**Table 6:** Estimates of heritability, direct genetic, maternal genetic, residual and phenotypic correlation between principal components and body weights, fitting best model for each trait using bivariate analysis

**PC**	**Body weight**	**Fitted model**	${{\varvec{h}}}_{1}^{2}$	${{\varvec{h}}}_{2}^{2}$	${{\varvec{c}}}_{1}^{2}$	${{\varvec{m}}}_{1}^{2}$	${{\varvec{m}}}_{2}^{2}$	${{\varvec{r}}}_{{\varvec{a}}}$	${{\varvec{r}}}_{{\varvec{m}}}$	${{\varvec{r}}}_{{\varvec{e}}}$	${{\varvec{r}}}_{{\varvec{p}}}$
PC1	BW	5, 3	0.15 ± 0.04	0.30 ± 0.05	0.02 ± 0.03	0.10 ± 0.03	0.13 ± 0.03	0.37 ± 0.14	0.83 ± 0.12	0.43 ± 0.04	0.45 ± 0.02
PC1	W360	5, 1	0.22 ± 0.05	0.29 ± 0.04	0.00 ± 0.02	0.06 ± 0.02		0.65 ± 0.08		0.73 ± 0.02	0.69 ± 0.01
PC2	BW	3, 3	0.12 ± 0.04	0.28 ± 0.05		0.09 ± 0.03	0.11 ± 0.03	0.64 ± 0.10	0.94 ± 0.07	0.69 ± 0.02	0.69 ± 0.01
PC2	W360	3, 1	0.13 ± 0.04	0.31 ± 0.04		0.10 ± 0.03		0.30 ± 0.16		-0.26 ± 0.04	-0.13 ± 0.02
PC3	BW	1, 3	0.09 ± 0.03	0.33 ± 0.05			0.10 ± 0.03	0.31 ± 0.17		0.08 ± 0.04	0.11 ± 0.02
PC3	W360	1, 1	0.08 ± 0.03	0.32 ± 0.04				0.37 ± 0.15		0.25 ± 0.03	0.26 ± 0.02

PC: principal component; fitted models are the models fitted for the PC and the body weights, respectively; BW and W360 are body weights (kg) at birth and 360 days of age, respectively. ${h}_{1}^{2}$ and ${h}_{2}^{2}$: direct heritabilities for PC and body weight, respectively; ${c}_{1}^{2}$: proportion of maternal permanent environmental variance to phenotypic variance for PC1; ${m}_{1}^{2}$ and ${m}_{2}^{2}$: maternal heritabilities for PC and body weight, respectively; ${r}_{a}$: direct genetic correlation; ${r}_{m}$: maternal genetic correlation; ${r}_{e}$: residual correlation; ${r}_{p}$: phenotypic correlation

Direct and maternal genetic, residual, and phenotypic correlations between principal components and parameters of the Brody and Richards functions are shown in Tables 7 and 8, respectively. PC1 had large positive direct genetic correlations with A for both functions (0.87 and 0.90) and a large negative direct genetic correlation with the degree of maturity associated with the maximum growth rate (u_I_) at the point of inflection of the Richards function (-0.80). Among the growth curve parameters, only u_0_ had a strong direct genetic relationship with PC2 (0.86 for the Brody function and 0.83 for the Richards function). In addition, a moderate but negative genetic correlation (-0.40) was estimated between PC2 and ${\text{u}}_{\text{I}}$ for the Richards function. Estimates of genetic correlations between PC1 and PC2 and the other growth-curve parameters were relatively small (|${r}_{a}$|≤ 0.29). Maternal genetic correlations between u_0_ and the first two PC were estimated to be very high and positive (≥ 0.89) for both growth functions. High to moderate negative genetic correlations of PC3 with k and u_0_ were obtained for both the Brody (-0.86 and -0.52, respectively) and Richards (-0.85 and -0.49, respectively) functions. In contrast, genetic relationships for PC3 with both A and t_50_ were high and positive for both growth functions (≥ 0.83). The additive genetic correlation between PC3 and u_I_ from the Richards function was likewise moderate and positive (0.59).

**Table 7:** Estimates of heritability, direct genetic, maternal genetic, residual and phenotypic correlations between principal components and Brody growth-curve parameters, fitting best model for each trait using bivariate analysis

**PC**	**Growth-curve parameter**	**Fitted models**	${{\varvec{m}}}_{1}^{2}$	${{\varvec{h}}}_{2}^{2}$	${{\varvec{c}}}_{1}^{2}$	${{\varvec{m}}}_{1}^{2}$	${{\varvec{m}}}_{2}^{2}$	${{\varvec{r}}}_{{\varvec{a}}}$	${{\varvec{r}}}_{{\varvec{m}}}$	${{\varvec{r}}}_{{\varvec{e}}}$	${{\varvec{r}}}_{{\varvec{p}}}$
PC1	A	5, 1	0.23 ± 0.05	0.19 ± 0.04	0.07 ± 0.03	0.01 ± 0.02		0.87 ± 0.07		0.42 ± 0.03	0.49 ± 0.02
	k	5, 1	0.20 ± 0.05	0.08 ± 0.03	0.07 ± 0.03	0.04 ± 0.03		-0.09 ± 0.22		0.30 ± 0.03	0.23 ± 0.02
	${u}_{0}$	5, 3	0.16 ± 0.04	0.13 ± 0.04	0.07 ± 0.04	0.07 ± 0.03	0.06 ± 0.02	-0.15 ± 0.22	0.89 ± 0.31	-0.18 ± 0.04	-0.10 ± 0.03
	${t}_{50}$	5, 1	0.22 ± 0.05	0.04 ± 0.02	0.05 ± 0.03	0.04 ± 0.03		0.10 ± 0.30		-0.39 ± 0.03	-0.31 ± 0.02
PC2	A	3, 1	0.13 ± 0.04	0.19 ± 0.04		0.10 ± 0.03		0.28 ± 0.19		-0.25 ± 0.04	-0.15 ± 0.02
	k	3, 1	0.12 ± 0.04	0.08 ± 0.03		0.10 ± 0.03		0.02 ± 0.25		0.07 ± 0.03	0.06 ± 0.02
	${u}_{0}$	3, 3	0.18 ± 0.05	0.14 ± 0.04		0.09 ± 0.03	0.03 ± 0.02	0.86 ± 0.06	0.99 ± 0.11	0.78 ± 0.01	0.80 ± 0.01
	${t}_{50}$	3, 1	0.12 ± 0.04	0.04 ± 0.02		0.10 ± 0.03		-0.08 ± 0.32		-0.06 ± 0.03	-0.05 ± 0.02
PC3	A	1, 1	0.09 ± 0.03	0.18 ± 0.04				0.90 ± 0.07		0.74 ± 0.01	0.75 ± 0.01
	k	1, 1	0.10 ± 0.03	0.07 ± 0.03				-0.86 ± 0.07		-0.89 ± 0.01	-0.88 ± 0.01
	${u}_{0}$	1, 3	0.09 ± 0.03	0.14 ± 0.04			0.07 ± 0.02	-0.52 ± 0.18		-0.47 ± 0.03	-0.46 ± 0.02
	${t}_{50}$	1, 1	0.07 ± 0.03	0.05 ± 0.03				0.83 ± 0.16		0.65 ± 0.02	0.66 ± 0.01

PC: principal component; fitted models are the models   fitted for the PC and growth-curve parameter, respectively;* A*: predicted final weight (kg); *k*: an indicator of relative growth rate (dW/dt)/*W* where W is weight (kg) at *t* days of age; ${u}_{0}$: degree of maturity at birth (*BW*/*A*); ${t}_{50}$: predicted age at 50% of *A;* ${h}_{1}^{2}$ and ${h}_{2}^{2}$: direct heritabilities for the PC and the growth-curve parameter, respectively; ${c}_{1}^{2}$: proportion of maternal permanent environmental variance to phenotypic variance for PC1; ${m}_{1}^{2}$ and ${m}_{2}^{2}$: maternal heritabilities for the PC and the growth-curve parameter, respectively; ${r}_{a}$: direct genetic correlation; ${r}_{m}$: maternal genetic correlation; ${r}_{e}$: residual correlation; ${r}_{p}$: phenotypic correlation

**Table 8:** Estimates of heritability, direct genetic, maternal genetic, residual and phenotypic correlations between principal components and Richards growth-curve parameters, fitting best model for each trait using bivariate analysis

**PC**	**Growth-curve parameter**	**Fitted models**	${{\varvec{h}}}_{1}^{2}$	${{\varvec{h}}}_{2}^{2}$	${{\varvec{c}}}_{1}^{2}$	${{\varvec{m}}}_{1}^{2}$	${{\varvec{m}}}_{2}^{2}$	${{\varvec{r}}}_{{\varvec{a}}}$	${{\varvec{r}}}_{{\varvec{m}}}$	${{\varvec{r}}}_{{\varvec{e}}}$	${{\varvec{r}}}_{{\varvec{p}}}$
PC1	A	5, 1	0.23 ± 0.05	0.20 ± 0.04	0.06 ± 0.02	0.01 ± 0.02		0.90 ± 0.06		0.51 ± 0.03	0.57 ± 0.02
	K	5, 1	0.20 ± 0.05	0.01 ± 0.02	0.08 ± 0.04	0.04 ± 0.03		-0.16 ± 0.57		0.11 ± 0.03	0.09 ± 0.02
	${u}_{0}$	5, 3	0.16 ± 0.04	0.11 ± 0.04	0.08 ± 0.04	0.06 ± 0.03	0.06 ± 0.02	-0.29 ± 0.22	0.99 ± 0.33	-0.26 ± 0.04	-0.17 ± 0.02
	${u}_{I}$	5, 1	0.19 ± 0.05	0.00 ± 0.02	0.08 ± 0.04	0.05 ± 0.03		-0.80 ± NE		0.01 ± 0.03	-0.01 ± 0.02
	${t}_{50}$	5, 1	0.20 ± 0.05	0.04 ± 0.03	0.06 ± 0.03	0.04 ± 0.03		0.14 ± 0.28		-0.32 ± 0.03	-0.25 ± 0.02
PC2	A	3, 1	0.13 ± 0.04	0.22 ± 0.04		0.10 ± 0.03		0.24 ± 0.18		-0.22 ± 0.04	-0.13 ± 0.02
	K	3, 1	0.13 ± 0.04	0.01 ± 0.02		0.10 ± 0.03		-0.29 ± 0.59		0.01 ± 0.03	0.00 ± 0.02
	${u}_{0}$	3, 3	0.18 ± 0.05	0.11 ± 0.04		0.10 ± 0.03	0.04 ± 0.02	0.83 ± 0.07	0.99 ± 0.10	0.79 ± 0.01	0.80 ± 0.01
	${u}_{I}$	3, 1	0.12 ± 0.04	0.01 ± 0.02		0.10 ± 0.03		-0.40 ± 0.95		0.06 ± 0.03	0.05 ± 0.02
	${t}_{50}$	3, 1	0.12 ± 0.04	0.04 ± 0.03		0.09 ± 0.03		-0.18 ± 0.30		-0.08 ± 0.03	-0.08 ± 0.02
PC3	A	1, 1	0.09 ± 0.03	0.21 ± 0.04				0.85 ± 0.09		0.58 ± 0.02	0.61 ± 0.02
	K	1, 1	0.08 ± 0.03	0.001 ± 0.02				-0.85 ± NE		-0.51 ± 0.02	-0.50 ± 0.02
	${u}_{0}$	1, 3	0.08 ± 0.03	0.13 ± 0.04			0.07 ± 0.02	-0.49 ± 0.20		-0.37 ± 0.03	-0.37 ± 0.02
	${u}_{I}$	1, 1	0.08 ± 0.03	0.00 ± 0.02				0.59 ± NE		-0.19 ± 0.03	-0.17 ± 0.03
	${t}_{50}$	1, 1	0.07 ± 0.03	0.05 ± 0.02				0.83 ± 0.16		0.65 ± 0.02	0.66 ± 0.01

PC: principal component; fitted models are the models   fitted for the PC and the growth-curve parameter, respectively; *A*: predicted final weight (kg); *k*: an indicator of relative growth rate (dW/dt)/*W* where W is weight (kg) at *t* days of age; ${u}_{0}$: degree of maturity at birth (*BW*/*A*); ${u}_{I}$: degree of maturity associated with the maximum relative growth rate; ${t}_{50}$: predicted age at 50% of *A;* ${h}_{1}^{2}$ and ${h}_{2}^{2}$: direct heritabilities for the PC and the growth-curve parameter, respectively; ${c}_{1}^{2}$: proportion of maternal permanent environmental variance to phenotypic variance for PC1; ${m}_{1}^{2}$ and ${m}_{2}^{2}$: maternal heritabilities for the PC and the growth-curve parameter, respectively; ${r}_{a}$: direct genetic correlation; ${r}_{m}$: maternal genetic correlation; ${r}_{e}$: residual correlation; ${r}_{p}$: phenotypic correlation; NE: not estimable

## Discussion

Variation in body weights at different ages in a population is explained by both variation among individuals at specific ages and longitudinal variation among individual growth curves. Growth in mammals is a complex phenomenon which is affected by several factors including genetically transmitted effects and maternal genetic potentials for nursing and caring for the offspring until weaning [4, 5]. The importance of these factors changes as the lambs age. For example, carryover maternal effects on body weight may be expressed after weaning but normally decline in importance with increasing lamb age, as the lambs become independent of the mother [6–8]. In this study, the standardized body weights of Muzaffarnagari lambs were distributed around zero, with an ascending trend in variation from birth to 12 months of age. Body weights were phenotypically correlated, especially at more advanced ages, but correlations decreased as the recording interval increased. Birth weight had a relatively small phenotypic correlation with the postnatal body weights, indicating that different genes may be influencing birth, compared to postnatal, weights, and that selection for birth weight may have only modest effects on postnatal growth.

Eigenvalues of the PC indicated that the marginal proportions of the total multivariate variation in body weight explained by each PC declined sharply from PC1 to PC5. More than 94% of the observed variation of body weight from birth to 12 months of age was explained by the first three PC, and these PC were considered adequate to explain multivariate variation in body weight in this breed. Similarly, the first three PC explained 81 and 70% of the variation in body size for Madura ewes and rams, respectively, and 69 and 67% of the variation in body size for ewes and rams, respectively, of the Rote breed [25]. These results presumably reflected the positive genetic, phenotypic, and residual correlations among body weights reported for most sheep breeds [26].

The PCA used a set of orthogonal vectors of body weights to allow characterization of lambs for contrasting aspects of the growth curves. The PCA weightings were empirically derived and provided ad hoc indicators of differences in growth. They therefore conceptually differed from estimates of theoretical growth curve parameters [23]. The eigenvector of weightings for PC1 placed similar levels of emphasis on each of the postnatal weights but less emphasis on birth weight, so that animals with higher values for PC1 had heavier postnatal weights. PC2 contrasted lambs that were heavier at birth but had smaller postweaning weights with those that had smaller birth weights and larger postweaning weights, potentially reflecting transmitted maternal genetic effects on prenatal growth that were not comparably expressed after birth. PC3 placed negative emphasis on 90-day weight and modest positive emphasis on weights recorded at ≥ 270 days of age.

Essentially identical weightings for PC1 through PC3 were obtained for lambs of different sexes or born in different seasons, indicating a high degree of consistency in the results of the PCA and facilitating use of PC as selection criteria. This result may have been unique to the semi-intensive management system applied in this study [23] and would not necessarily be repeated for lambs raised under more extensive management conditions, especially, in tropical and low- to medium-input systems, where environmental factors have a greater influence on growth trajectories. Our findings suggest greater consistency of PC compared to estimates of growth curve parameters, which have been reported by several authors to be affected by environmental factors [3, 17, 23, 27, 28]. On the other hand, given the estimates for maternal heritabilities for the PC and their pattern of associations with body weight, the PC would be suitable selection criteria to improve both the overal growth rate and curve parameters of Muzaffarnagari sheep under the prevalent production system.

Maternal genetic and permanent environmental effects significantly affected PC1 with c^2^ = 0.08 and m^2^ = 0.04, but PC2 was influenced solely by maternal genetic effects. Only direct genetic effects were significant for PC3. As expected, both genetic and phenotypic variances decreased from PC1 to PC3, with direct heritabilities from the best model of 0.19, 0.12, and 0.08, respectively. The total maternal effect (m^2^ + c^2^) was similar for PC1 (0.12) and PC2 (0.10), in agreement with the value of m^2^ = 0.10 for birth weight in bivariate analysis with PC3. These findings confirmed that genetic variation in PC2 mainly reflected direct and maternal genetic variation in birth weight.

The PC were, by definition, phenotypically independent, but were genetically positively correlated, with the estimates ranging from 0.19 between PC2 and PC3 to 0.48 between PC1 and PC2 and 0.72 between PC1 and PC3 (Table 5). A very high positive maternal genetic correlation of 0.97 between PC1 and PC2 was also present. These results suggest that, despite the phenotypic independence among the PC, their underlying genetic relationships remained consistent with the strong positive inter-correlations among sequential body weights in this breed [29, 30]. Selection based on the PC will therefore be less effective in modifying the realized growth curves than would be expected based on their observed phenotypic independence. Potential appears to exist to modify birth weights without associated major changes in subsequent postnatal weights, as evidenced by the modest genetic correlation of 0.48 between PC1 and PC2 but could be limited by the maternal genetic correlation of 0.97. Application of growth curve analysis to these data [23] indicated that the asymptotic Brody function was superior to several other three-parameter functions for describing growth in this population. The four-parameter Richards function provided a more flexible model of sigmoidal growth but was not superior to the Brody function in goodness of fit and may have overparameterized the growth function relative to the available data. Both functions included estimates of an asymptotic final body weight (A), a maturing-rate parameter (k), and a constant of integration (B) that defines the degree of maturity at birth (u_0_ = birth weight /A). The Richards function also has a shape parameter (m) that defines the degree of maturity at the inflection point (u_I_) of the Richards function as u_I_ = [(m-1)/m]^m^ [23].

The growth curve parameter A is a primary driver of body size and had phenotypic correlations with PC1 of 0.49 for the Brody function (Table 7) and 0.57 for the Richards function (Table 8). PC2 was mainly controlled by variation in birth weight, with r_p_ of 0.80 with u_0_ for both growth functions. Remaining correlations of PC1 and PC2 with estimates of growth curve parameters were low (|r_p_|≤ 0.31). At the phenotypic level, PC3 was associated with both large adult size and late maturity, with r_p_ of 0.75 with A, -0.88 with k and -0.46 with u_0_ for the Brody function. These correlations were consistent in sign but somewhat lower in magnitude for the estimates of parameters of the Richards function, confirming previous evidence that these functions have similar genetic architecture [2]. Phenotypic correlations involving PC3 were consistent with the negative weightings for early postnatal body weights and positive weightings for later body weights in PC3, suggesting that negative selection based on PC3 may have potential to modify the growth curve, resulting in smaller final weights but more rapid maturing rates. However, PC3 accounted for only 8% of the multivariate phenotypic variance in body weights. The phenotypic SD of PC3 was only 0.62 compared to 0.88 for PC2 and 1.87 for PC1, and the heritability of PC3 was lower than those of PC2 and PC1 (0.08 vs. 0.12, and 0.19, respectively). The potential for negative selection on PC3 to change the shape of the growth curve was therefore limited by relatively low genetic variance.

The direct genetic correlation of PC1 with A exceeded 0.86 for both growth functions and was larger than the genetic correlation of 0.65 between PC1 and yearling weight. Selection on PC1 would therefore be expected to increase all of the postnatal body weights and generate a strong positive response in A. A high positive genetic correlation of 0.86 and 0.83 obtained between PC2 and u_0_ from Brody and Richards curves, respectively, suggested a potential to improve the degree of maturity at birth by selecting for PC2. This result was further supported by the estimate of ${r}_{a}$ = 0.64 between PC2 and the actual birth weight. A negative genetic correlation was reported between A and u_0_ [2], but PC1 and PC2 were positively genetically correlated. Additionally, a strong positive maternal genetic correlation of PC2 with u_0_ (r_m_ ≥ 0.99) for both functions as well as with the observed birth weight (r_m_ ≥ 0.94) indicated that selection for PC2 would improve maternal genetic breeding values for traits related to the birth weight.

In bivariate models with the PC, maternal effects on estimates of growth curve parameters were observed only for u_0_, with values of m^2^ ranging from 0.03 to 0.07. Significant maternal effects were not observed for any of the growth curve parameters that described the postnatal growth trajectory from birth to maturity (i.e., k, t_50_, or, for the Richards function, u_I_) [2]. This result contrasts with previous multitrait analyses of lamb body weights that generally identified significant additive maternal and (or) ewe permanent environmental effects on (pre)weaning weights. These maternal effects commonly declined gradually and become non-significant at advanced postweaning ages. A review of genetic parameters for sheep reported average values of 0.11 for m^2^ and 0.08 for c^2^ for lamb weaning weights [26]. The semi-intensive management imposed in the current data may have limited expression of maternal effects on body weight, but there is little support for such a result in the literature. Analyses of data from the U.S. National Sheep Improvement Program yielded estimates of (m^2^ + c^2^) of 0.19 and 0.13 for (pre)weaning weights recorded at 60 and 120 days of age, respectively, in Targhee sheep [31], and estimates of (m^2^ + c^2^) of 0.17 for weaning weights recorded at 42 to 125 d of age in Katahdin sheep [32]. Data from the same recording system yielded estimates of (m^2^ + c^2^) of 0.23, 0.16, 0.19, and 0.05 for (pre)weaning weights at 30, 60, 90, and 120 days of age, respectively, in Suffolk sheep, and 0.32, 0.22, and 0.23 for (pre)weaning weights at 30, 60, and 90 days of age, respectively, in Polypay sheep [33]. Suffolk and Polypay lambs would have been substantially more likely than Targhee and Katahdin lambs to have been housed and intensively creep fed during the preweaning period, indicating that the maternal contribution to lamb weaning weight was not larger for more extensively managed sheep breeds.

Inability of growth curve analyses to identify maternal effects on early lamb growth (e.g. on k, t_50_, or u_I_) [2] provided some of the motivation for the current study, in order to determine if PCA would assist in detection of maternal genetic effects. However, this goal was at best marginally realized. Significant effects of m^2^ and c^2^ were observed for PC1, indicating the presence of maternal effects on lamb body weights across all ages. Maternal effects were observed for PC2 but were essentially identical to those observed for birth weight or u_0_. PC3 differentially weighted early and late body weights in a manner consistent with a maternal influence on early growth but was not significantly influenced by maternal effects, perhaps due to inability to accurately partition the much lower phenotypic variation observed for PC3 compared to PC1 and PC2.

Results of the current study, when combined with those of Mandal et al. [2, 23, 29, 30], can be used to consider alternative selection criteria for growth in Muzaffarnagari lambs. A multitrait mixed model evaluation provides a baseline for genetic evaluation and is facilitated in the current data by considering only five body weights recorded at specific ages. A multitrait analysis also permits use of correlations among recorded weights to account for missing data and early culling of candidates for selection. Use of multitrait analyses to produce a vector of multitrait estimated breeding values (EBV) for each lamb provides a relatively complete depiction of the realized growth trajectory given the level of body weight recording [29]. However, if the frequency of recording and the number of body weights (n) increases, the computational demands of the multi-trait analysis increase rapidly. The number of elements in the left-hand side matrix (LHS) of the mixed-model equations equals $\left[{\left(p+q\right)}^{2}\times {n}^{2}\right]$ where p and q are the levels of fixed and random effects, respectively. In particular, use of automated body weight recording technologies can lead to very large vectors of body weights, with variable potential for missing weights. In such a situation, the use of estimates of growth curve parameters limits n to the number of parameters for the growth function but requires preliminary processing of individual body weight vectors to estimate the growth-curve parameters for each lamb. Estimation of individual growth curve parameters is possible in the presence of some variation among lambs in the ages at recording, but records are generally required across the full recording space. Early termination of body weight recording may result in changes in growth curve parameter estimates, so accounting for early removal of lambs, due to death or culling, is not straightforward. Estimates of the growth parameter A for ewe lambs based on records of lamb weights through 202 or 365 days of age, respectively, were considerably less than the observed adult ewe body weights [11, 23]. If preliminary culling occurs at a few specific times, a return to a multitrait approach could be applied but would require that parameter estimates with different ending ages be treated as different traits and would increase the dimension of LHS. Use of PCA shares many of the advantages of growth curve analysis, but yields realized ad hoc, rather than theoretical, growth descriptors.

A key limitation of classical PCA is that it requires complete data for all the lambs, which presents challenges for practical implementation of PC-based selection criteria to improve growth traits in sheep, goats, and beef cattle. This challenge arises due to the common practice of culling non-replacement animals, including most males and a portion of female progenies, before reaching maturity. Consequently, body weight records at later ages are unavailable for a substantial number of animals. In the current study, the original dataset comprised 16,202 body weight records from 4480 lambs. However, body weight records across all time points required for PC computation were only available for approximately half of these animals (n = 2234) prior to data editing and quality control. This limitation is reflective of a broader issue common in datasets collected for grazing livestock species, as per our experiences. Strategies for imputation of missing values in PCA exist [34, 35], but further increase preliminary computing requirements. Despite these constraints, the study's findings demonstrated a high degree of consistency in PC weightings across seasons and sexes, which underscore the potential utility of PC-based approaches. However, it is important to evaluate the consistency across fixed-effect levels for each population, and specially commercial flocks, to ensure the broader applicability of these findings. Therefore, we recommend further investigations of the effectiveness of PC-based methodologies in commercial production systems and their applicability in sheep breeding programs, using larger datasets from commercial populations.

Aside from issues involved in computation of EBV, use of indicators of lamb growth in selection also requires integration of the derived EBV into some sort of breeding objective, with assignment of index weightings to each body weight or derived parameter estimate. This is a nontrivial task for a series of body weights. The EBV weightings are normally defined as partial derivatives of some goal function holding all other EBV constant. For a set of multitrait EBV, deciding on, for example, the economic value of body weight at 270 days holding genetic merit for all of the other weights constant is challenging. For even modest values of n, a simplified breeding objective will often be desired (and perhaps necessary) to define a practical breeding goal. For example, in the current study, birth, weaning, and yearling weights could appear in the breeding objective, but with all five recorded weights used to estimate their EBV. Use of EBV for estimates of growth curve parameters is theoretically attractive and could be achieved using simulation to determine the impact of each parameter on some bioeconomic goal trait (e.g., [36]). Use of the PC as breeding objectives is more difficult because different vectors of individual body weights can potentially yield the same value for an individual PC. Direct simulation of, for example, the effect of increasing breeding values for PC3 holding PC1 and PC2 constant is correspondingly challenging.

In the current study, selection for average body weight from birth to 12 months of age, for yearling weight, for growth curve parameter A, or for PC1 would all be expected to increase additive direct and maternal genetic potential for body weight at all postnatal ages. Likewise, selection for birth weight, u_0_, or PC2 would all increase direct and maternal additive effects on birth weights with only modest increases in postnatal weights. However, selection to change the shape of the growth curve from birth to maturity would be more challenging. Selection for maternal genetic effects on early postnatal body weights should enhance early growth rates, potentially modifying the phenotypic growth curve. However, relatively low heritabilities for k and very low heritabilities for t_50_ in the Brody curve and very low heritabilities for k, u_I_, and t_50_ in the Richards function suggest little opportunity to modify the growth trajectory from birth to 12 months of age. Negative selection for PC3 appears to have some potential to modify the growth curve, but limited phenotypic variation and failure to identify additive maternal effects would limit the predicted selection response.

## Conclusions

The first three principal components considered the multivariate variation of body weight of Muzaffarnagari sheep from different aspects and angles, with a high degree of consistency for lambs of different sexes or born in different seasons. These features facilitate use of PC as alternative selection criteria in sheep breeding programs. When compared to results of multitrait genetic analyses of either individual body weights or estimates of growth curve parameters for each lamb, PC1 and PC2 provided easily interpretable indicators of general body size and birth weight, respectively. However, use of principal components to modify the growth trajectory from birth to maturity was challenging. Negative selection on PC3 had potential to modify the shape of the growth curve by restricting increases in yearling weight while increasing the maturing rate but would have limited effect because of low genetic variation in PC3.

## Supplementary Information

Additional file 1. Figure S1. Cumulative proportion of explained variance (CPEV) and proportion of explained variance (PEV) of the principal components for body weight from birth through 12 months of age in Muzaffarnagari sheep by lamb sex. Eigenvalues, which reflect the importance of each principal component in explaining the multivariate variation in body weight, are shown at the top of each column. Figure S2. Cumulative proportion of explained variance (CPEV) and proportion of explained variance (PEV) of the principal components for the body weight at birth to yearling in Muzaffarnagari sheep by birth season. Eigenvalues, which reflect the importance of each principal component in explaining the multivariate variation in body weight, are shown at the top of each column. Figure S3. Pearson correlations between the principal components (PC) and the adjusted body weights at birth (BW) and at 90 (W90), 180 (W180), 270 (W270), and 360 (W360) days of age. Figure S4. Eigenvectors of the principal components for the body weighs at birth (BW) and at 90 (W90), 180 (W180), 270 (W270), and 360 (W360) days of age by lamb sex. Figure S5. Eigenvectors of the principal components for the body weighs at birth (BW) and at 90 (W90), 180 (W180), 270 (W270), and 360 (W360) days of age by birth season.

## Data Availability

The authors affirm that all data necessary for confirming the conclusions of the article are present within the article, figure and tables.
