# Supplementary material for: Principal components-based selection criteria for genetic improvement of growth in sheep breeding programs
Source: Genet Sel Evol. 2025 Sep 25;57:49. doi: 10.1186/s12711-025-00992-0 (PMC12465440; doi:10.1186/s12711-025-00992-0)
Supplement: Supplementary file 1 — Additional file 1. Figure S1. Cumulative proportion of explained variance (CPEV) and proportion of explained variance (PEV) of the principal components for body weight from birth through 12 months of age in Muzaffarnagari sheep by lamb sex. Eigenvalues, which reflect the importance of each principal component in explaining the multivariate variation in body weight, are shown at the top of each column. Figure S2. Cumulative proportion of explained variance (CPEV) and proportion of explained variance (PEV) of the principal components for the body weight at birth to yearling in Muzaffarnagari sheep by birth season. Eigenvalues, which reflect the importance of each principal component in explaining the multivariate variation in body weight, are shown at the top of each column. Figure S3. Pearson correlations between the principal components (PC) and the adjusted body weights at birth (BW) and at 90 (W90), 180 (W180), 270 (W270), and 360 (W360) days of age. Figure S4. Eigenvectors of the principal components for the body weighs at birth (BW) and at 90 (W90), 180 (W180), 270 (W270), and 360 (W360) days of age by lamb sex. Figure S5. Eigenvectors of the principal components for the body weighs at birth (BW) and at 90 (W90), 180 (W180), 270 (W270), and 360 (W360) days of age by birth season. [file 12711_2025_992_MOESM1_ESM.pdf]

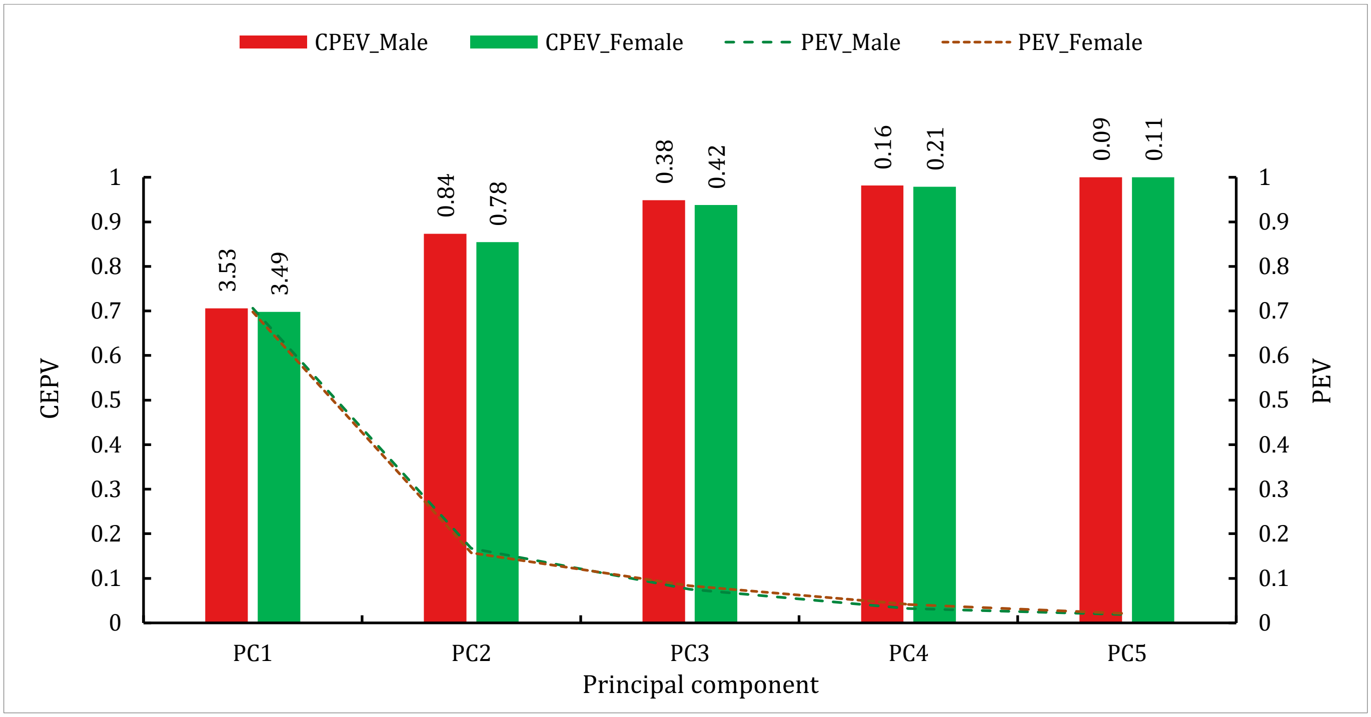

Figure S1: Cumulative proportion of explained variance (CPEV) and proportion of explained variance (PEV) of the principal components for body weight from birth through 12 months of age in Muzaffarnagari sheep by lamb sex. Eigenvalues, which reflect the importance of each principal component in explaining the multivariate variation in body weight, are shown at the top of each column.

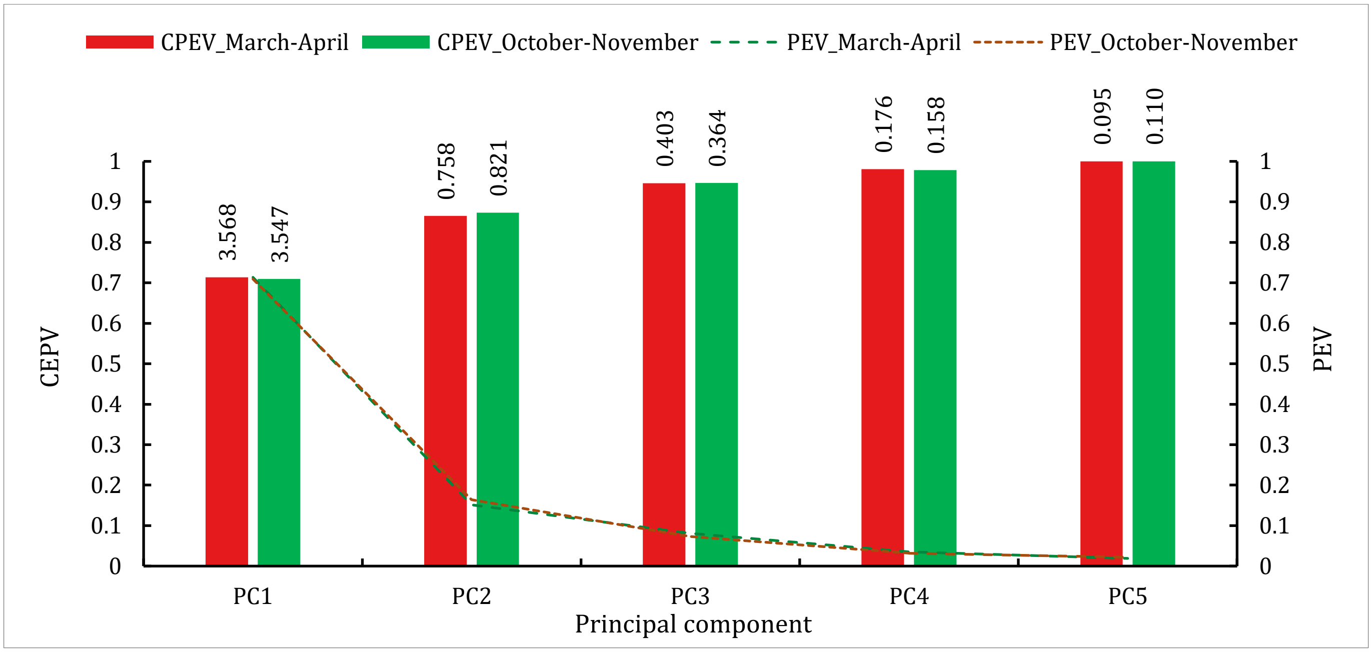

Figure S2: Cumulative proportion of explained variance (CPEV) and proportion of explained variance (PEV) of the principal components for the body weight at birth to yearling in Muzaffarnagari sheep by birth season. Eigenvalues, which reflect the importance of each principal component in explaining the multivariate variation in body weight, are shown at the top of each column.

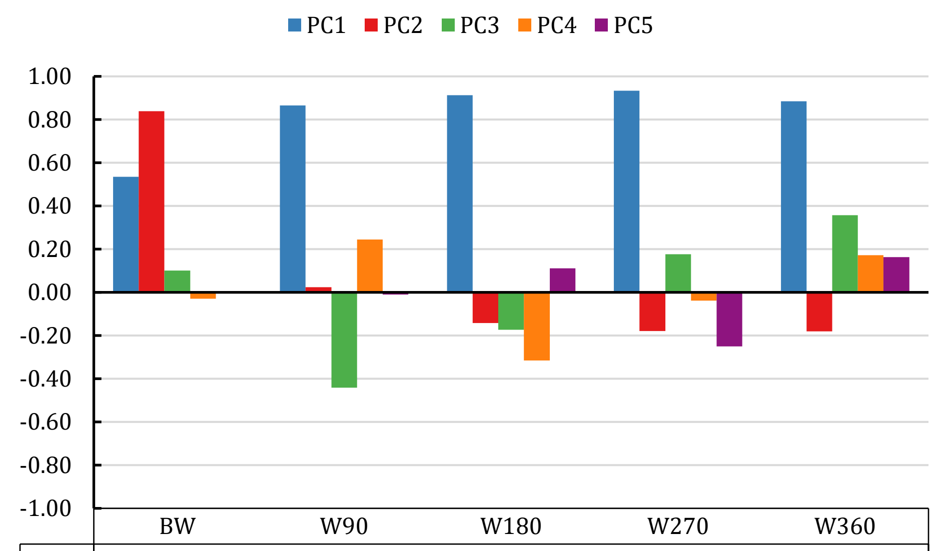

	BW	W90	W180	W270	W360
PC1	0.54	0.87	0.91	0.93	0.89
PC2	0.84	0.02	-0.14	-0.18	-0.18
PC3	0.10	-0.44	-0.17	0.18	0.36
PC4	-0.03	0.25	-0.31	-0.04	0.17
PC5	0.00	-0.01	0.11	-0.25	0.16

Figure S3. Pearson correlations between the principal components (PC) and the adjusted body weights at birth (BW) and at 90 (W90), 180 (W180), 270 (W270), and 360 (W360) days of age.

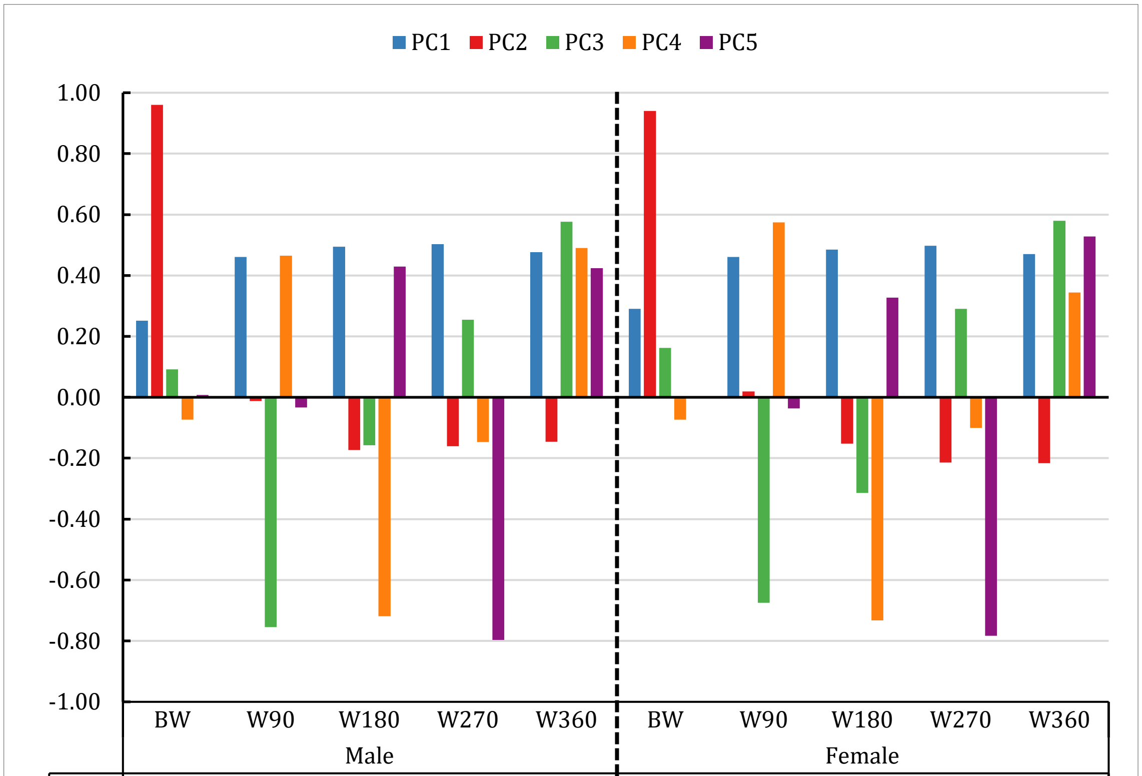

	Male BW	Male W90	Male W180	Male W270	Male W360	Female BW	Female W90	Female W180	Female W270	Female W360
PC1	0.25	0.46	0.49	0.50	0.48	0.29	0.46	0.48	0.50	0.47
PC2	0.96	-0.01	-0.17	-0.16	-0.15	0.94	0.02	-0.15	-0.21	-0.22
PC3	0.09	-0.75	-0.16	0.25	0.58	0.16	-0.68	-0.31	0.29	0.58
PC4	-0.07	0.47	-0.72	-0.15	0.49	-0.07	0.57	-0.73	-0.10	0.34
PC5	0.01	-0.03	0.43	-0.80	0.42	0.00	-0.04	0.33	-0.78	0.53

Figure S4: Eigenvectors of the principal components for the body weighs at birth (BW) and at 90 (W90), 180 (W180), 270 (W270), and 360 (W360) days of age by lamb sex.

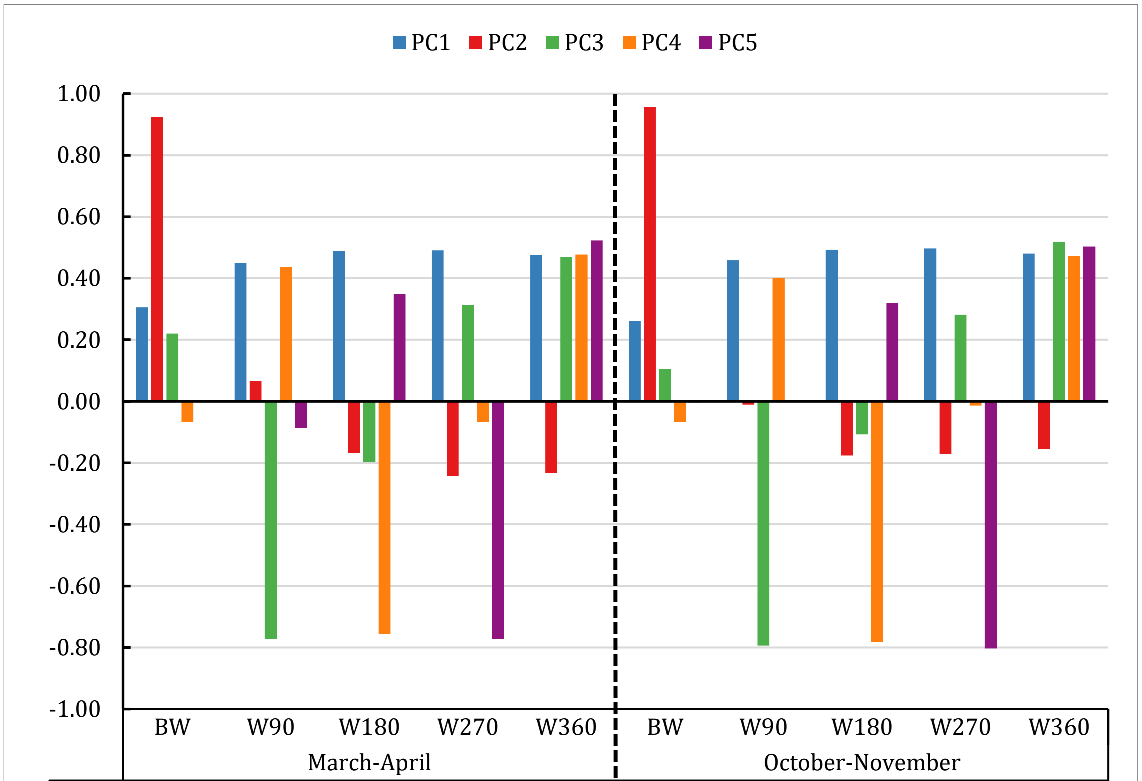

	March-April					October-November				
	BW	W90	W180	W270	W360	BW	W90	W180	W270	W360
PC1	0.31	0.45	0.49	0.49	0.47	0.26	0.46	0.49	0.50	0.48
PC2	0.92	0.07	-0.17	-0.24	-0.23	0.96	-0.01	-0.18	-0.17	-0.15
PC3	0.22	-0.77	-0.20	0.31	0.47	0.11	-0.79	-0.11	0.28	0.52
PC4	-0.07	0.44	-0.76	-0.07	0.48	-0.07	0.40	-0.78	-0.01	0.47
PC5	0.00	-0.09	0.35	-0.77	0.52	0.00	0.00	0.32	-0.80	0.50

Figure S5: Eigenvectors of the principal components for the body weighs at birth (BW) and at 90 (W90), 180 (W180), 270 (W270), and 360 (W360) days of age by birth season.
